# Supplementary material for: The Genus Capsicum: A Review of Bioactive Properties of Its Polyphenolic and Capsaicinoid Composition
Source: Molecules. 2023 May 22;28(10):4239. doi: 10.3390/molecules28104239 (PMC10224380; doi:10.3390/molecules28104239)
Supplement: Supplementary file 1 [file molecules-28-04239-s001.zip › molecules-2369001-supplementary.pdf]

Figure S1

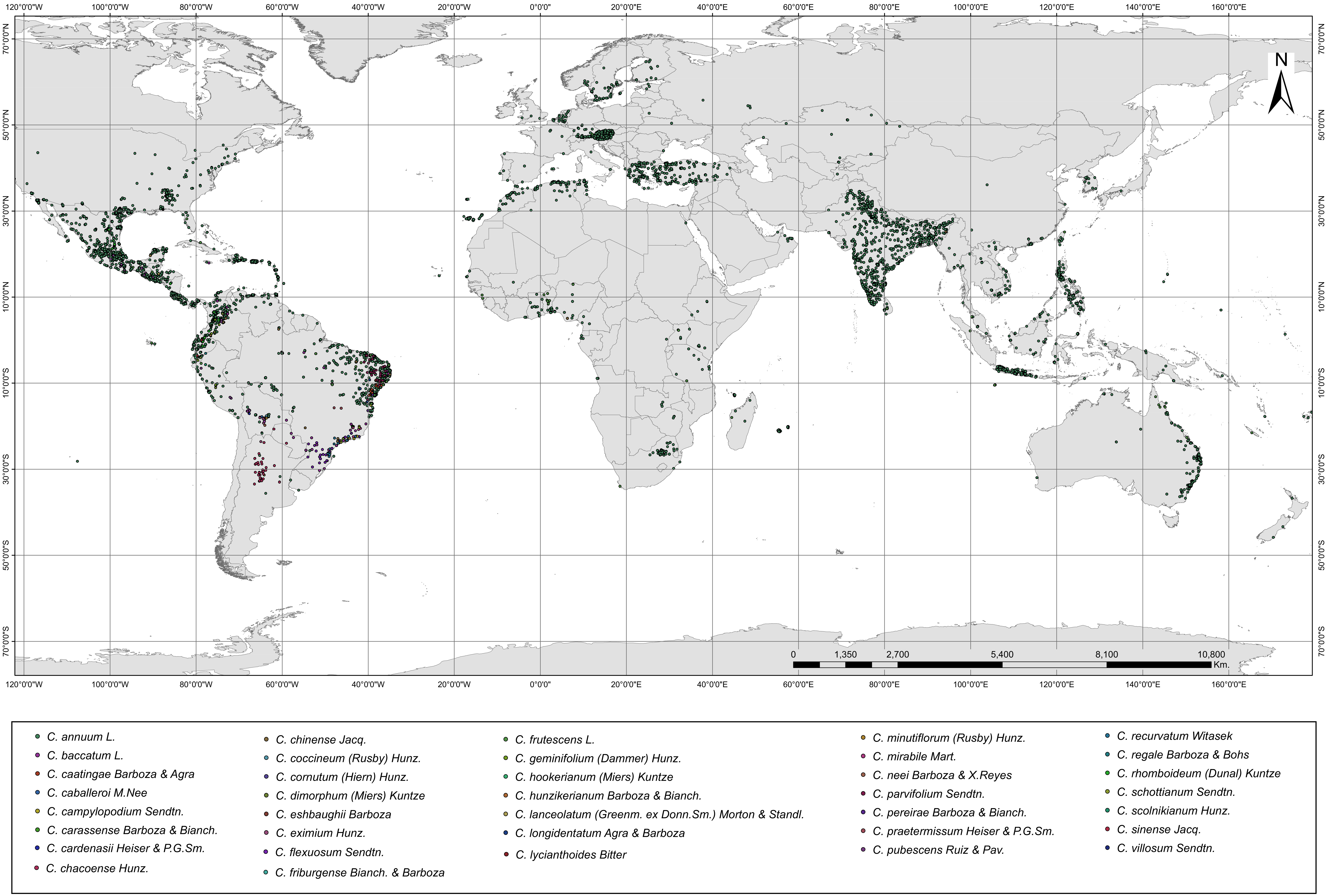
120°0'0"W
100°0'0"W
80°0'0"W
60°0'0"W
40°0'0"W
20°0'0"W
0°0'0"
20°0'0"E
40°0'0"E
60°0'0"E
80°0'0"E
100°0'0"E
120°0'0"E
140°0'0"E
160°0'0"E
70°0'0"N
50°0'0"N
30°0'0"N
10°0'0"N
10°0'0"S
30°0'0"S
50°0'0"S
70°0'0"S
N
0
1,350
2,700
5,400
8,100
10,800
Km.
C. annuum L.
C. baccatum L.
C. caatingae Barboza & Agra
C. caballeroi M.Nee
C. campylopodium Sendtn.
C. carassense Barboza & Bianch.
C. cardenasii Heiser & P.G.Sm.
C. chacoense Hunz.
C. chinense Jacq.
C. coccineum (Rusby) Hunz.
C. cornutum (Hiern) Hunz.
C. dimorphum (Miers) Kuntze
C. eshbaughii Barboza
C. eximium Hunz.
C. flexuosum Sendtn.
C. friburgense Bianch. & Barboza
C. frutescens L.
C. geminifolium (Dammer) Hunz.
C. hookerianum (Miers) Kuntze
C. hunzikerianum Barboza & Bianch.
C. lanceolatum (Greenm. ex Donn.Sm.) Morton & Standl.
C. longidentatum Agra & Barboza
C. lycianthoides Bitter
C. minutiflorum (Rusby) Hunz.
C. mirabile Mart.
C. neei Barboza & X.Reyes
C. parvifolium Sendtn.
C. pereirae Barboza & Bianch.
C. praetermissum Heiser & P.G.Sm.
C. pubescens Ruiz & Pav.
C. recurvatum Witasek
C. regale Barboza & Bohs
C. rhomboideum (Dunal) Kuntze
C. schottianum Sendtn.
C. scolnikianum Hunz.
C. sinense Jacq.
C. villosum Sendtn.
